# The Reproducibility of 31-Phosphorus MRS Measures of Muscle Energetics at 3 Tesla in Trained Men

**DOI:** 10.1371/journal.pone.0037237

**Published:** 2012-06-11

**Authors:** Lindsay M. Edwards, Damian J. Tyler, Graham J. Kemp, Renee M. Dwyer, Andrew Johnson, Cameron J. Holloway, Alan M. Nevill, Kieran Clarke

**Affiliations:** 1 Department of Physiology, Anatomy and Genetics, University of Oxford, Oxford, United Kingdom; 2 The Oxford Centre for Clinical Magnetic Resonance Research, John Radcliffe Hospital, Oxford, United Kingdom; 3 Department of Musculoskeletal Biology, Faculty of Health and Life Sciences, University of Liverpool, Liverpool, United Kingdom; 4 School of Medicine, University of Tasmania, Hobart, Australia; 5 School of Sport, Performing Arts and Leisure, University of Wolverhampton, Wolverhampton, United Kingdom; University of Las Palmas de Gran Canaria, Spain

## Abstract

**Objective:**

Magnetic resonance spectroscopy (MRS) provides an exceptional opportunity for the study of *in vivo* metabolism. MRS is widely used to measure phosphorus metabolites in trained muscle, although there are no published data regarding its reproducibility in this specialized cohort. Thus, the aim of this study was to assess the reproducibility of ^31^P-MRS in trained skeletal muscle.

**Methods:**

We recruited fifteen trained men (VO_2_peak = 4.7±0.8 L min^−1^/58±8 mL kg^−1^ min^−1^) and performed duplicate MR experiments during plantar flexion exercise, three weeks apart.

**Results:**

Measures of resting phosphorus metabolites were reproducible, with 1.7 mM the smallest detectable difference in phosphocreatine (PCr). Measures of metabolites during exercise were less reliable: exercising PCr had a coefficient of variation (CV) of 27% during exercise, compared with 8% at rest. Estimates of mitochondrial function were variable, but experimentally useful. The CV of PCr_1/2t_ was 40%, yet much of this variance was inter-subject such that differences of <20% were detectable with *n* = 15, given a significance threshold of *p*<0.05.

**Conclusions:**

31-phosphorus MRS provides reproducible and experimentally useful measures of phosphorus metabolites and mitochondrial function in trained human skeletal muscle.

## Introduction

Magnetic resonance spectroscopy (MRS) is unmatched in its ability to measure tissue biochemistry in intact humans without the need for invasive procedures or the administration of potentially harmful radioactive isotopic tracers. In particular, it has been used extensively to monitor 31-phosphorus (^31^P) metabolites in both cardiac [1] and skeletal muscle [2]. Due to the large volume and easy accessibility of the skeletal muscles of the human leg, ^31^P-MR spectra can be acquired from a localized volume of leg muscle with excellent temporal (>1/s) resolution. Thus ^31^P-MRS can be used to measure steady-state concentrations of high-energy phosphorus metabolites in resting skeletal muscle and phosphorus metabolite kinetics during exercise and recovery in a single experiment. It has long been known that the kinetic constants during work transitions provide an insight into the energy metabolism of the exercising (and recovering) muscle (cf [3]). Therefore resting phosphorus metabolites, and their kinetics during transitions from exercise to rest, have been widely used to assess muscle energetic status and energy metabolism, both in healthy subjects [4], [5], [6], [7], [8] and in patients with a wide range of diseases [9], [10], [11], [12], [13]. Indeed, in many cases MRS may well provide the only accurate *in vivo* measure of metabolites with rapid turnover in humans and experimental animals.

There have been two recent reports on the reproducibility of ^31^P-MRS measurements in healthy untrained human skeletal muscle [14], [15]. These recent papers added to an existing body of work using a range of experimental approaches that are summarized in Table 1. Results from these diverse approaches have been quite consistent in showing that ^31^P-MRS is generally very reproducible, although one of the more comprehensive studies [14] seemed to suggest that estimates of mitochondrial function (made using kinetic data) are less so, at least compared with measurements of resting phosphocreatine concentration. In addition, the reproducibility studies that have been conducted using repeated testing in a single subject [16], [17], [18], although helpful in uncovering measurement or intra-individual variability, are unable to detect either systematic bias or population-dependent (inter-individual) variability.

**Table 1:** Summary of published data regarding the reproducibility of ^31^P-magnetic resonance spectroscopy in skeletal muscle (in chronological order).

Experimental design	Timing	Cohort	Exercise modality/muscle group	Reference
Multiple test-retest	20 minutes between tests	1 healthy subject	Isometric/thumb	Miller et al. (1987) [17]
Single test-retest	1 month between tests	1 healthy female subject	Isometric/calf	Miller et al. (1995) [16]
Multiple test-retest	‘two different days’	4 moderately active subjects	Dynamic/calf	Walter et al. (1997) [41]
Single test-retest	1 month between tests	7 healthy untrained females	Isometric/calf	Larson-Meyer et al. (2000) [37]
Single test-retest	1 week between tests	18 sedentary males (‘who complained of fatigue’)	Dynamic/finger	Bendahan et al. (2002) [42]
Multiple test-retest	24 hours	14 children	Dynamic/thigh	Barker et al. (2006) [43]
Multiple test-retest	N/a	1 healthy subject	Dynamic/thigh	Van den Broek et al. (2007) [18]
Multiple test-retest	1 month/1 year between tests	11 untrained males+1 untrained female	Dynamic/thigh	Layec et al. (2009) [14]
Multiple test-retest	‘1–30 days’ between tests	12 healthy untrained males	Isometric/thigh	McCully et al. (2009) [15]

Investigators in other fields have found that there are differences (both improvements and decrements) in the reproducibility of experimental methods when applied to exercise-trained subjects as opposed to untrained controls [19], [20]. As with sedentary or moderately active subjects, ^31^P-MRS is widely used to measure phosphorus metabolites and kinetics in the muscles of trained subjects, yet Table 1 shows that there are no published data reporting directly on the reproducibility of the method in this specialized cohort. However, what data there are suggest that both the inter- and within-subject variability of ^31^P-MRS indices of mitochondrial function may differ markedly in athletes; for example, recently published data suggest that the coefficients of variation of several estimates of mitochondrial oxidative rate differ more than sevenfold between sedentary and endurance-trained subjects [21]. Thus, the aim of this study was to assess the reproducibility of MRS measures of 31-phosphorus metabolism in trained human skeletal muscle. We hypothesized that, despite differences in oxidative capacity between a trained and an untrained cohort, ^31^P-MRS would continue to provide reliable, repeatable and useful measures of muscle biochemistry *in vivo*.

## Methods

### Ethics Statement

The Central Oxfordshire Research Ethics Committee approved this study and fully-informed written consent was obtained from all subjects. All protocols were conducted in accordance with the Declaration of Helsinki.

**Figure 1:**
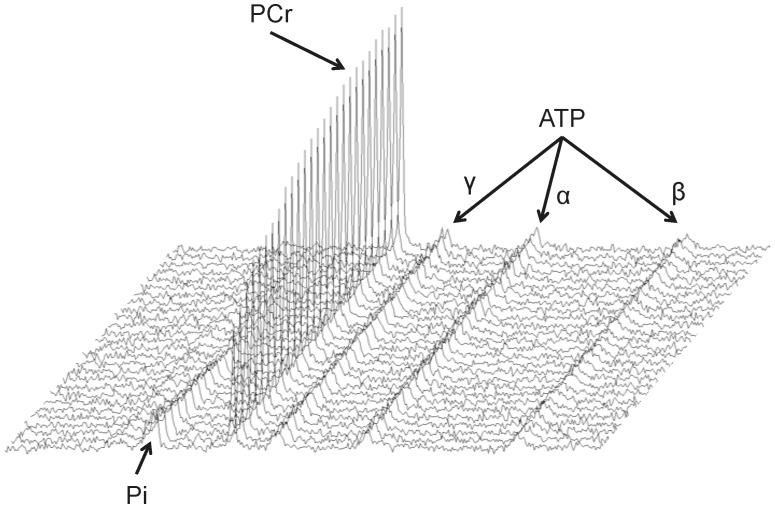
Stacked plot showing 31-phosphorus magnetic resonance spectra acquired at 5-second intervals from the calf muscle of a single trained subject in recovery from dynamic exercise.

These data were acquired as part of a larger study. We recruited fifteen trained men from the Oxford rowing crews. We chose rowers for our study based on their participation in an aerobic sport that requires significant recruitment of the plantar flexion muscles of the lower leg [22]. Standard MR contraindications were excluded by history and physical examination. Peak aerobic capacity (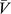
O_2_peak) was measured as described in detail elsewhere [23], [24]. Ventilatory threshold was calculated according to the V-slope method [25], using software supplied for use with the Metamax system (Metasoft 3, Cortex, Biophysik, Germany). Subsequent MR experiments, the details of which have been published elsewhere [23], [24], [26], were performed twice, three weeks apart. Subjects were instructed to maintain normal training patterns for the two weeks prior to each measurement. Each subject performed plantar flexion exercise in a Siemens Trio 3T clinical MR system (Siemens, Erlangen, Germany), with a 6 cm dual-tuned ^31^P and ^1^H surface coil placed under the widest part of the right gastrocnemius. A special wooden housing was constructed to ensure that coil positioning was consistent and repeatable. Positioning was further refined through the use of scout images. Prior to the acquisition of ^31^P MR time-series data, three baseline scans were acquired to allow calculation of correction factors for partial saturation due to the short repetition time (TR) in the main acquisition, and for nuclear Overhauser enhancement (NOE). The acquisition parameters for the ^31^P time-series were TR 500 ms, TE 0.35 ms, bandwidth 2000 Hz, 10 averages, 512 data points, excitation flip angle 25° and 10 rectangular NOE pulses, with pulse duration 10 ms, inter-pulse delay 10 ms and excitation flip angle 180°. The MR exercise protocol was: 5 min rest, 5 min very light exercise (warm-up), 7 min recovery, 5 min at 5 W, 7 min recovery, 5 min at 6 W, 5 min recovery. Exercising values are the means of the last minutes of bouts 2 and 3. Figure 1 shows a typical set of spectra, acquired at 5-second intervals during the recovery phase.

**Figure 2:**
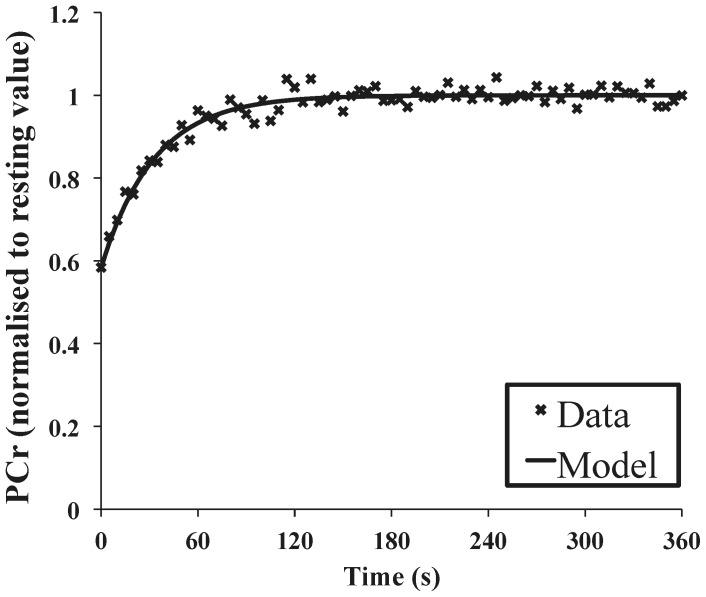
Typical experimental data (phosphocreatine concentration, normalised to resting values, in recovery from dynamic exercise) and a monoexponential function (solid line), fitted as described in Methods.

Spectra were processed using jMRUI version 2.2 [27] and quantified using a non-linear least squares algorithm [28]. The resting ATP concentration was taken as 8.2 mM [2]. The chemical shift of the inorganic phosphate (Pi) peak, relative to phosphocreatine (PCr), was used to determine intracellular pH. Intracellular [ADP] was calculated making the standard assumption that the creatine kinase reaction was at equilibrium, and correcting for pH [29]. The halftime of PCr recovery after moderate exercise (PCr_t1/2_) was determined by fitting a monexponential equation to the PCr recovery data. Figure 2 shows a typical fit to experimental data. The maximum rate of mitochondrial ATP synthesis (Q_MAX_) was extrapolated from the end-exercise [ADP] and corresponding rate of PCr resynthesis as in [30]. Technical issues caused a loss of data for calculation of Q_MAX_ in a single subject. Thus *n* = 14 for this and associated measurements.

Statistical analyses were conducted using PASW 18.0 (SPSS Inc., Chicago, USA). Reproducibility was assessed using techniques drawn from [31] and [32]. Heteroscedasticity was treated as significant if the correlation between the means of the repeated measures and the absolute difference between them was positive and significant at *p*<0.05. In these cases, data were log transformed. A paired *t*-test was used to assess test-retest bias. The standard deviation of the differences was taken as an index of test-retest variability. In addition to these traditional methods, 95% confidence intervals of the differences between means were calculated. In the case of heteroscedastic data, 95% confidence intervals were calculated for the log-transformed data. When ‘antilogged’ these confidence limits are ratios, and are reported as such. In the main text, data are reported as means (SD).

**Table 2:** Subjects’ characteristics (*n* = 15).

Age (y)	22 (1)
Mass (kg)	82 (9)
Absolute VO2peak (L min^−1^)	4.7 (0.8)
Relative VO2peak (mL min^−1^ kg^−1^)	58 (8)
Ventilatory threshold (%)	75 (12)

## Results

The subjects (*n* = 15) were aged 22 (1) years, weighed 82 (9) kg (Table 2). They had a peak aerobic capacity of 4.7 (0.8) L min^−1^ (58 (8) mL min^−1^ kg^−1^) and a ventilatory threshold of 75 (12) % of peak power, confirming their trained status.

**Figure 3:**
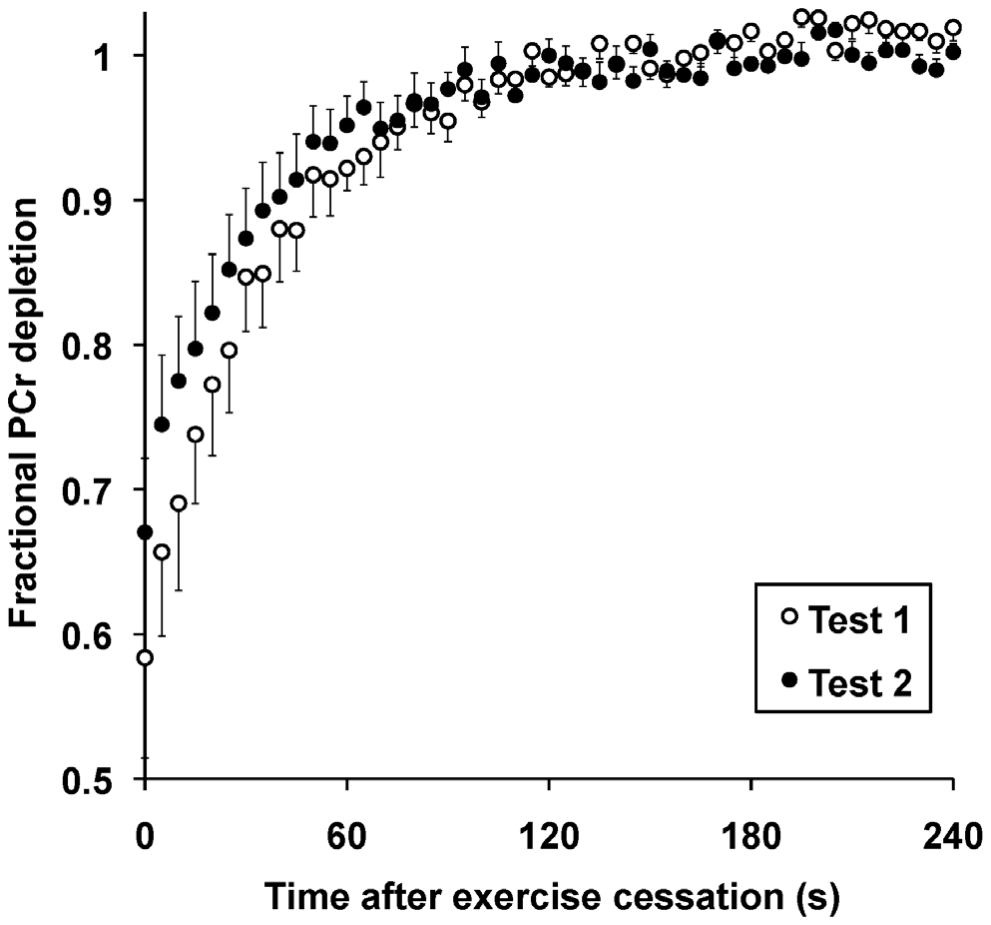
Phosphocreatine (PCr) recovery (normalised to resting values) in trained human calf muscle after dynamic exercise during two separate but identical tests. Values shown are means±SEM.

Table 3 summarises the results of our analysis, giving the means and standard deviations of the first and second measures in each case, accompanied by the grand coefficient of variation (CV) where applicable. For example, muscle phosphocreatine content was measured as 30 (3) mM on the first visit and 29 (2) mM on the second; the CV for this measurement was 8%. Figure 3 shows the group means (and standard errors) for phosphocreatine concentration in recovery from dynamic exercise.

**Table 3:** Reproducibility of ^31^P-MRS in trained skeletal muscle (*n* = 15).

				Means vs. absolute differences		Bias (mean diff.)	Error (SD of diff.)	95% confidence limits of the difference	
Parameter (*n* = 15)	Mean 1 (SD)	Mean 2 (SD)	CV (%)	*r*	*p*			Lower	Upper
Mean resting [PCr] (mM)	30 (3)	29 (2)	8	0.22	0.43	−1	3	−2.9	0.5
Mean exerc. [PCr] (mM)	18 (5)	18 (4)	27	−0.27	0.34	1	4	−1.6	2.8
Mean resting [Pi] (mM)	4 (1)	4 (1)	17	0.04	0.90	−0.1	0.9	−0.6	0.4
Mean exerc. [Pi] (mM)	13 (7)	14 (6)	47	**0.53**	**0.04** *	1	4	×0.94	×1.24
Ln (exerc. [Pi])	2.5 (0.5)	2.6 (0.4)	N/a	−1.0	0.73	0.1	0.3	−0.1	0.3
Mean resting pH	7.06 (0.02)	7.06 (0.02)	0.2	−0.17	0.55	0.01	0.02	−0.01	0.02
Mean exerc. pH	7.00 (0.08)	6.99 (0.08)	1	−0.36	0.19	−0.01	0.07	−0.05	0.03
Mean resting [ADP] (µM)	24 (8)	28 (7)	29	0.03	0.92	4	10	−2	9
Mean exerc. [ADP] (µM)	72 (24)	64 (20)	32	0.28	0.31	−9	25	−23	5
PCr_1/2t_ (sec)	23 (9)	19 (8)	40	0.18	0.53	−4	6	−7.06	−0.02
Q_MAX_ (µM s^−1^)^1^	405 (158)	427 (119)	33	**0.55**	**0.04** *	22	175	×0.85	×1.4
Ln (Q_MAX_)^1^	5.9 (0.4)	6.0 (0.3)	N/a	0.02	0.95	0.1	0.4	−0.16	0.33

* Significant correlation between means and absolute differences at p<0.05. ^1^n = 14.

Table 3 also shows the results of our tests of heteroscedasticity, as recommended by Nevill and Atkinson [32]. In two cases (exercising [Pi] and Qmax) there was convincing evidence of heteroscedasticity (i.e. a significant positive correlation between the absolute magnitude of the difference between two observations and their mean). These data were log-transformed and tested for heteroscedasticity again. In both cases the heteroscedasticity was resolved.

We looked for test-retest bias (for example, instrument drift or a learning effect) using a paired *t*-test comparing the first and second measurements. Table 3 shows that there was no significant test-retest bias in any of the measures taken. The standard deviation of the differences between the first and second measures (‘Error (SD of diff.)’ in Table 3) is an index measurement variability (as described in detail by Bland and Altman [31]). We extended this approach by calculating the 95% confidence intervals for the differences between the first and second measures. These confidence intervals give the minimum limits for the detection of changes at a significance threshold of *p*<0.05. For example, in our trained cohort of fifteen, an increase in resting muscle [PCr] of >0.5 mM or a decrease of >2.9 mM would have been significant at the *p*<0.05 level. In the case of log-transformed data these confidence limits were antilogged to provide a 95% confidence ‘ratio’. For example, in our cohort an increase in exercising [Pi] of >24% or a decrease of >6% would have been significant at *p*<0.05.

In all cases the 95% confidence intervals were not symmetrical due to nonsignificant bias. If one assumes that bias was not present (as the data suggest) then the confidence intervals can be corrected. Thus a change in resting muscle [PCr] of ±1.7 mM ((0.5+2.9)/2) could be reasonably assumed to be detectable at *p*<0.05 using our methods and with *n* = 15. Likewise, the minimum detectable change in exercising [Pi] would be ±15%.

## Discussion

We studied the reproducibility of ^31^P-MRS indices of muscle metabolism in a trained cohort, for the first time (to our knowledge). We found that measures of resting metabolites were the most repeatable, with CVs of 8% (PCr) and 17% (Pi). Exercising metabolites were more variable (27% (PCr) and 47% (Pi)). Finally, measures of mitochondrial function such as PCr_1/2t_, while highly variable (CV = 40%) were still experimentally useful providing a relative detection threshold of <20% (*n* = 15, *p*<0.05).

Training (and recovery) stimulates adaptive physiological changes that vary widely in their timing. Thus it seems reasonable to suggest that the coefficients of variation of a range of physiological parameters measured in athletes may be different to those in sedentary subjects. This hypothesis has led researchers in other areas to specifically study the effect of exercise training on the reproducibility of various experimental methods [19], [20]. Bingisser *et al.* ^19^ found that there were significant differences in reproducibility between measures taken in trained vs. untrained subjects, with the trained subjects being more homogenous and thus more reproducible in the measures that were studied. Likewise, Heitkamp and colleagues ^20^ studied the reproducibility of the lactate threshold in trained vs. untrained women. Once again, measurements in the trained women were somewhat more reliable.

Among the many well-known adaptive changes that follow from high levels of physical activity, exercise training stimulates changes in muscle gene transcription [33]. This may explain why muscle oxidative enzyme activity can vary widely in trained or highly-active humans compared with those who are sedentary [34], and why the coefficients of variation of ^31^P-MRS estimates of mitochondrial function can differ markedly in athletes compared to controls [21]. Furthermore, within trained subjects the peripheral training effect can vary dramatically even at the same relative VO_2_ [35]. Consistent with this, the coefficients of variation (CV) we observed in our trained cohort were larger than those reported in untrained subjects [14]. For example, the CV of resting [PCr] in our trained cohort was 8%, compared with 2.2% reported by Layec *et al.* [14] and ∼5% by Roussel and co-workers [36]. Yet resting muscle pH, which one would not expect to vary with training status, had a very similar CV in our trained cohort vs. earlier studies in untrained subjects: the CV of resting muscle pH was 0.2% in our hands and was reported as being 0.28% by Layec *et al.* [14], 0.4% by Roussel *et al.* [36] and 0.1% by Larson-Meyer and colleagues [37]. Given that the calculation of muscle pH from ^31^P-MRS data utilises two independent peaks in a single spectrum, this comparability between the two studies reinforces that our data were of a similar quality to those earlier studies.

Yet despite the slightly greater variation, ^31^P-MRS in athletes had excellent reproducibility when measuring intramuscular phosphates. In the absence of significant bias, the smallest detectable difference for a given *n* can be estimated from the mean of the absolute values of the confidence intervals (as outlined in Results). Using this approach, we estimate that changes in [PCr] of ∼2.1 mM (7%) could be detected in just 10 trained subjects.

Consistent with earlier studies, measures of mitochondrial function were more variable. Coefficients of variation in our trained subjects were >30% for both PCr_1/2t_ and Q_MAX_. This is compared to coefficients of variation of ∼20% for PCr_1/2t_ [14], [15] 13–30% for Qmax [14] in other studies. However, the measurement of PCr_1/2t_ in athletes is unfairly described by these statistics. Although there was a high degree of inter-individual variation, analysis of the differences (measurement 2– measurement 1) suggested that changes of <20% could be detected in 15 trained subjects, an eminently feasible number for practical research, particularly given that endurance trained individuals have a Q_MAX_ that is close to double that of untrained individuals [38] and exercise training can induce increases in mitochondrial function of the order of up to 50% in the untrained elderly [39]. The reliability of measurements of metabolite concentration during exercise lay between those same measurements at rest and the indices of mitochondrial function (Table 3). The increased variation relative to resting measurements could be attributed to several sources: First, despite heavy strapping and careful experimental design, noise may been generated due to motion/contraction of the target muscles. In addition, variations in aerobic fitness/mitochondrial function and, possibly, ATP-economy of contraction were likely to have contributed to increased variance [40].

One could argue that the lack of tight control over our subjects’ training schedules led to increased variability. However, our aim was to assess reproducibility in this cohort under ‘normal’ conditions (i.e. without strict training control). Nevertheless, the lack of any evidence for increased variability suggests that tight controls may be unnecessary during magnetic resonance studies of athletes.

There were three potential sources of variability in our data: variability in the instrument, physiological variation and processing variability (for example, slight differences in the selection of data used for curve fitting). Earlier studies have addressed these issues by i. Duplicate acquisitions from the same subject under identical conditions (i.e. in immediate succession, cf. [17]), ii. Repeated measurements on the same individual at different times (as in the present study) and iii. Duplicate processing of the same data by the same experimenter on different occasions (as in [14]). The existing work suggests that instrument variability and processing variability contribute rather little to the overall variability. Thus it seems reasonable to suggest that the bulk of the variability we observed was physiological in nature. However, these three sources of variability are difficult to separate entirely (for example, a given instrument may operate with greater variability across several days or months, but no living biological matrix is unchanging across these timescales). For the present study we chose not to separate these sources of variation as, in practice, they are all present; our aim was to produce benchmark data regarding the reliability of the method as a whole. One must consider that our study used athletes whose training was not being directly controlled by the experimenters. As such, variations in training load or the timing of experimental acquisition relative to training sessions may have introduced greater variability than in a cohort where training was rigorously controlled.

To conclude, we studied the reproducibility of ^31^P-MRS measures of muscle phosphorus metabolism in a cohort of trained men. The coefficients of variation in this cohort appear to be slightly larger than in earlier, similar studies that used untrained subjects. However, these larger coefficients of variation appeared to be the result of larger inter-individual variation, while test-retest reliability remained good. Thus we found the method to be reproducible and reliable enough for studies to be conducted using relatively small numbers of trained participants, especially where paired statistical comparisons will be used.
